# The last flight of F/O Tadeusz Stabrowski. Identification of the polish pilot

**DOI:** 10.3389/fgene.2023.1231451

**Published:** 2023-07-26

**Authors:** Dagmara Lisman, Joanna Drath, Iwona Teul, Grażyna Zielińska, Maria Szargut, Joanna Dowejko, Sandra Cytacka, Jarosław Piątek, Jan Ambroziak, Grzegorz Śliżewski, Andrzej Ossowski

**Affiliations:** ^1^ Department of Genetic Forensic, Pomeranian Medical University, Szczecin, Poland; ^2^ Polish Ministry of Heritage and National Culture, Warsaw, Poland; ^3^ Polish Aviation Historical Foundation, Warsaw, Poland

**Keywords:** STR markers, Y-STR, polish pilot, identification, NN victims

## Abstract

The paper presents the process of identifying an unnamed soldier of the Polish armed forces in the west, whose remains were found in a nameless grave at the municipal cemetery in Le Crotoy in France. The Polish Genetic Database of Victims of Totalitarianism team carried out the research in cooperation with the Ministry of Culture and National Heritage. A comprehensive analysis of autosomal and Y-STR markers was performed. Historical, anthropological, and forensic examinations of the remains were also carried out. The items found with the remains were also examined. Identification based on DNA analysis made it possible to restore the identity of the Polish pilot who died on 11 March 1943 near the French coast, F/O Tadeusz Stabrowski. The airman regained his name in 2018, he was about 26 years old at the time of his death and left behind a grieving wife and son in the United Kingdom. The success of identifying the NN remains was guaranteed by the appointment of an interdisciplinary team consisting of specialists in archaeology, anthropology, history, forensic medicine and forensic genetics. The analysis of historical sources allowed to determine 4 missing airmen whose remains could have been buried in the cemetery in Le Crotoy. An interesting aspect of the research was the cooperation with history enthusiasts and fans of Polish aviation, thanks to which it was finally possible to narrow down the group of pilots sought and reach the family of Tadeusz Stabrowski, who submitted comparative material for research. This is the first case of establishing the identity of a Polish pilot killed in France. Many institutions have been involved in the project, including Polish Ministry of Culture and National Heritage (MDiKN), which partially funded the research.

## 1 Introduction

In August 1941, the No. 308 “City of Kraków” Polish Fighter Squadron RAF, a unit of the Polish Air Force in Great Britain, lost two men in combat flights and one man in an aerial accident. The squadron cultivated the traditions first introduced by the pilots of the Fighter Squadron of the second Air Regiment in Krakow (September 1939). The airmen flew many flights over occupied France and Benelux countries. S/Ldr Marian Pisarek (commander of the Cracovians) was appointed in cooperation with the squadron’s branch. In this way, Tadeusz Stabrowski joined forces and arrived on September 24 in Northolt, where the 308th Fighter Squadron was stationed. This unit is part of one of the three squadrons of the first Polish Fighter Wing (Northolt Wing) flying from outside occupied Europe. These units were then famous for being the best and most effective in the RAF. His first combat flight Tadeusz Stabrowski in the squad of 308 DM made on October 2, it was a sweep over the English Channel. It was then that F/O was assigned on September 24th. He has participated in many successful assignments (Król, 1982; Zdzisław Sawicki, 2007).

On 11 March 1943, F/O Tadeusz Stabrowski, together with Sgt Stanisław Domański, undertook an attack on ground targets in northern France. During this action, his plane was hit by anti-aircraft artillery. Despite this, F/O Stabrowski decided to return to England. On the way, about 10 miles off the coast of France, smoke started coming out of the Spitfire, forcing it to ditch. He got out of the cockpit of the plane. According to the testimony of his companion (Sgt Domański), he was floating on the surface of the sea. The sea rescue plane that arrived on the scene tracked the target, but the crew lost sight of it during landing. Low water temperature and, consequently, hypothermia probably contributed to his death. His body was found on 12 April 1942, on a beach a few kilometers north of Le Crotoy (La voie de Rue). He was buried as an unnamed pilot in St. Firmin at Le Crotoy (Koliński, 1978; Król, 1982).

A breakthrough was brought by the exhumation and genetic research carried out in 2017 by the team of the Polish Genetic Database of Victims of Totalitarianism of the PUM in cooperation with the MDiKN. It was known that at the time of his death, he was married and left behind a less than a 6-month-old son. It was he, a 75-year-old man, who was found in Canada and provided comparative material for genetic research. A huge role in finding Tadeusz Stabrowski’s family was played by the private e-mail contact of the Polish historian Ph.D. Grzegorz Śliżewski, who had contact with Tadeusz Stabrowski’s wife a few years earlier, was already dead at the time of the search. Reaching his son was difficult for many reasons, including the fact that he moved from Great Britain to Canada and lived there for several decades, and an act that he did not use the surname Stabrowski, but the surname of his stepfather who raised him. The identification was preceded by a thorough analysis of historical materials and the selection of an NN airman who was buried in a French cemetery.

Thanks to the involvement of MDiKN, it was possible to reach the relatives of the selected pilots and obtain reference material from them. In this way, four pilots who could be buried at the Le Crotoy cemetery were indicated: Józef Gil, Leon Kosmowski, Jerzy Skibiński, and Tadeusz Stabrowski (Table 1).

**TABLE 1:** Missing pilots taking into consideration as potential persons who could be buried in Le Crotoy.

Name of missing	Army unit	Date of birth	Date of go missing	Family info
F/O J.G	306 Fighter Squadron	30.01.1914	31.12.1942	available
P/O L.K	306 Fighter Squadron	31.03.1919	31.12.1942	available
P/O J.S	610 Fighter Squadron	15.11.1919	13.02.1943	available
P/O Tadeusz Stabrowski	308 Fighter Squadron	16.05.1917	11.03.1943	available

**FIGURE 1:**
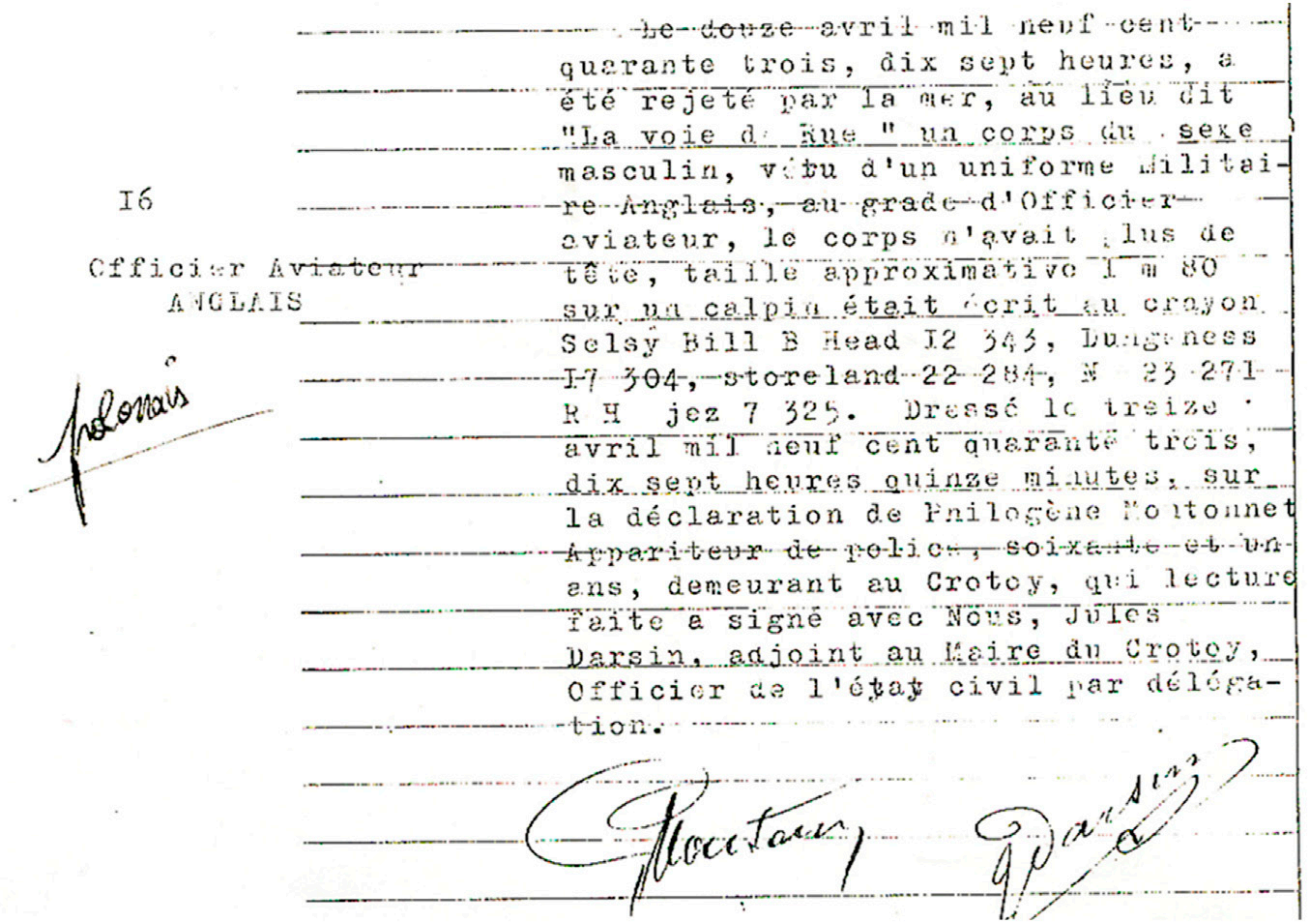
Photo of a document drawn up by the French Police after finding the remains of an unnamed person, the document provided by the Ministry of Culture and National Heritage.

The Ministry of Culture and National Heritage also submitted a document drawn up by the French Police after finding the remains of an unnamed person for research (Figure 1).

The document states: „On 12 April 1943, at 5 p.m., a male body was thrown over the sea in a place called “La voie de Rue”, dressed in an English military uniform, the rank of an air force officer, the body had no head, body height about 1 m80 on the card was written in pencil Sesly Bill B Head I2 343, Dungeness I7 304, Storeland 22 284, N 23 271, RH lake 7 325. Buried at the request of Philogene Montonnet Police Representative, age 61, buried in Crotoy.” On the side, there is a description I 6 Lotnik English and a note of Pole. (Figure 1).

The exhumation work began on 28 September 2017. The administration of the cemetery in Le Crotoy removed the tomb stele and elements of the tomb the day before. 50 cm of the earth was removed, revealing the outline of the grave pit, on this basis, the exact location of the burial was determined. The remains rested at a depth of about 75 cm. It has been established that the skeleton was originally placed in a wooden coffin. At the time of burial, the body must have been heavily decomposed, as evidenced by the arrangement of the remains. The remains were arranged only partially in anatomical order. Numerous items of equipment of a British pilot from the Second World War were also revealed.

## 2 Objective of the work

The main aim of the work was to properly collect and analyze historical, anthropological, forensic, and genetic data obtained during the analysis of exhumed remains at the French cemetery in Le Crotoy.

## 3 Material and methods

### 3.1 Evidence material

**FIGURE 2:**
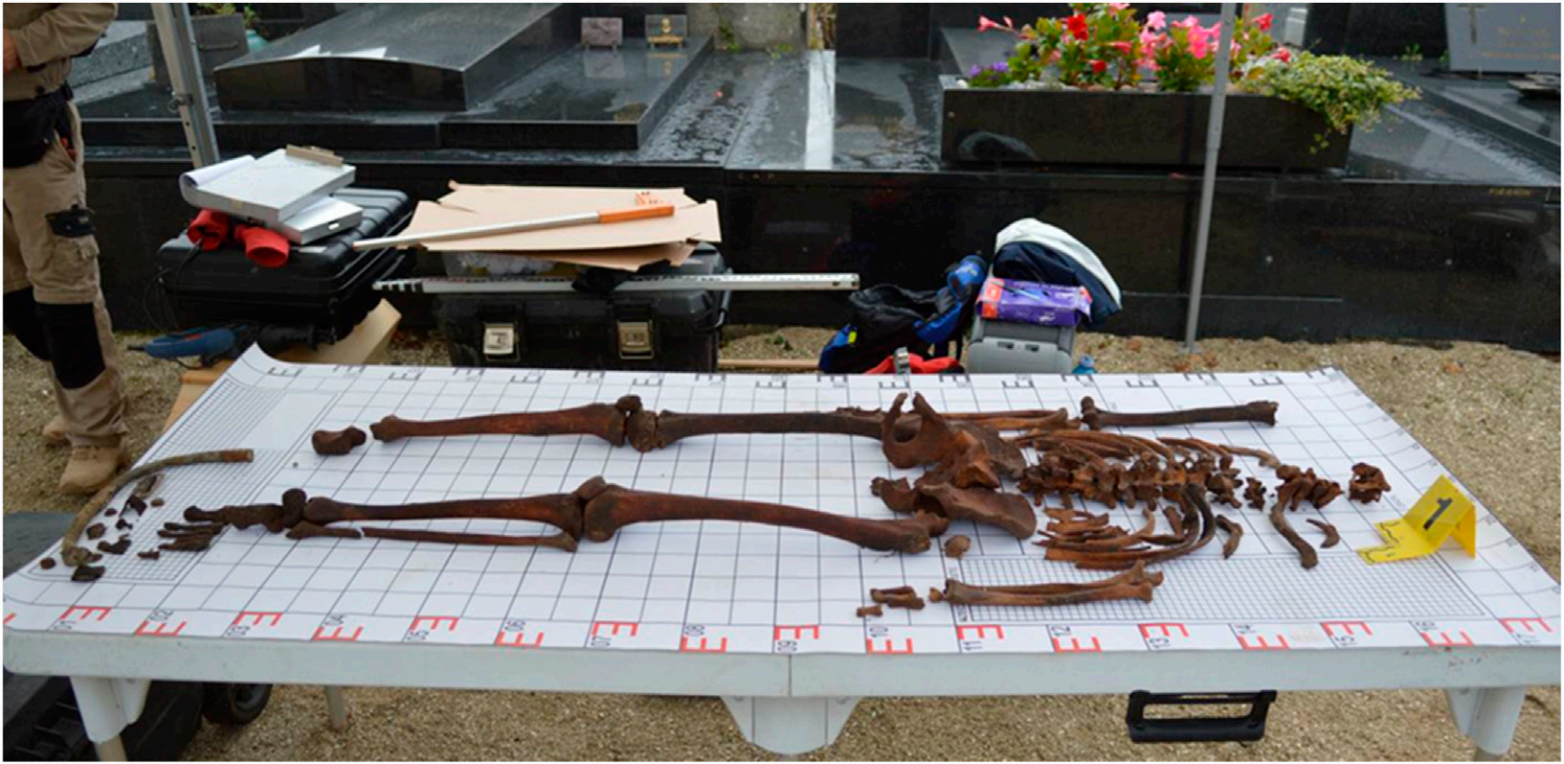
Remains taken from the grave pit and placed on the autopsy table—preliminary anthropological analyses.

**FIGURE 3:**
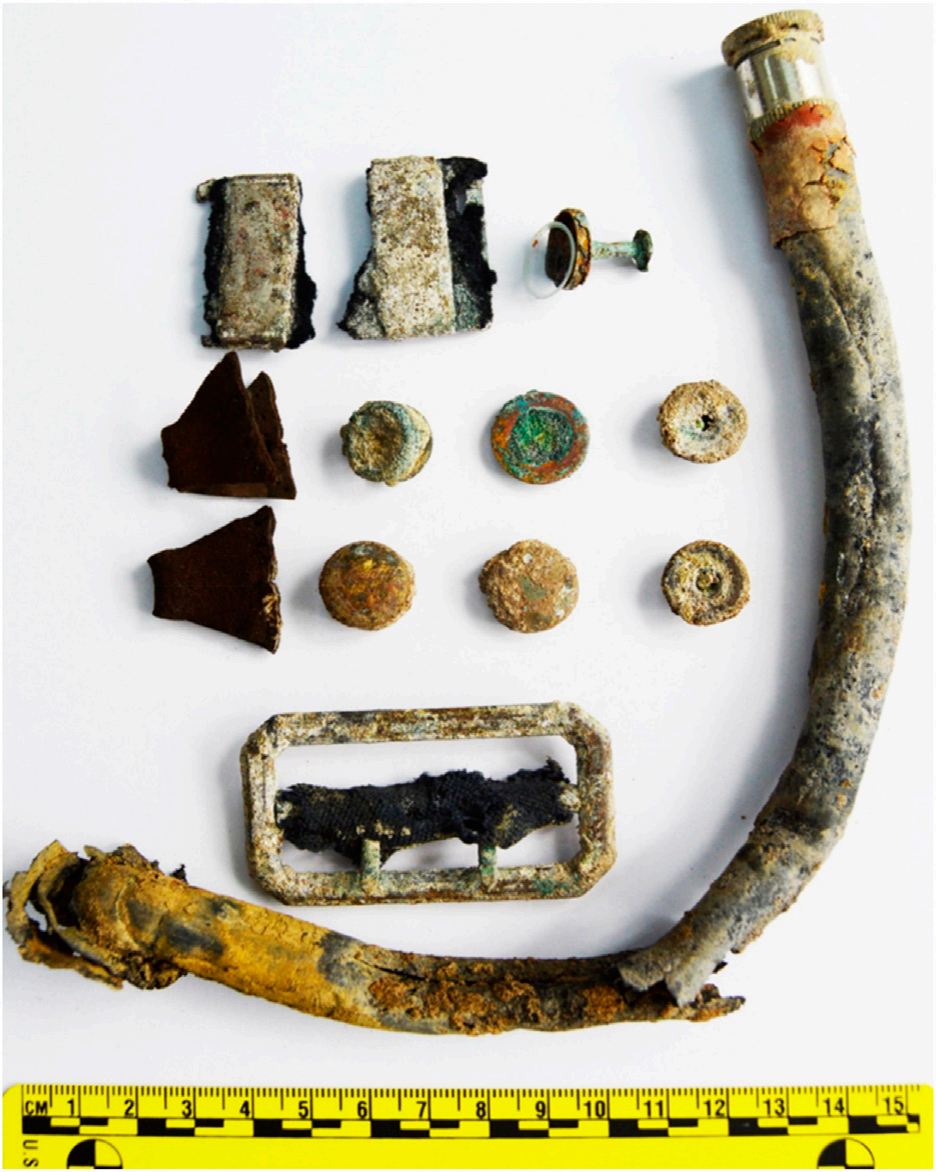
Artifacts found in the grave pit—latches, a fragment of a rubber hose with a fitting from an RAF pilot’s life jacket, and an escape compass.

**FIGURE 4:**
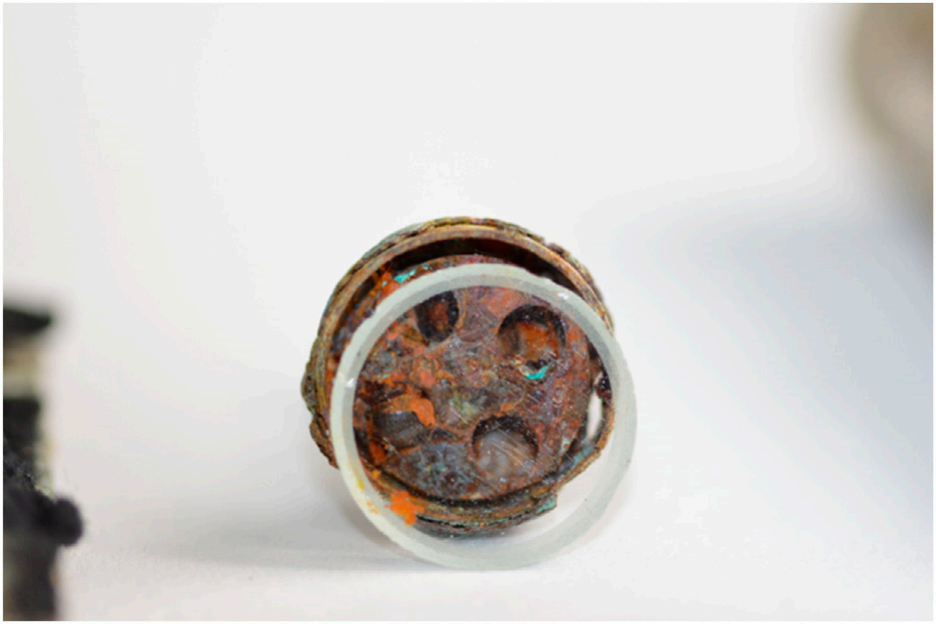
Escape compass fund with the remains of Tadeusz Stabrowski.

The research material was a skeleton exhumed on 28 September 2017, at the French cemetery in Le Crotoy (Figure 2). The remains rested at a depth of about 75 cm, in a partial anatomical arrangement, they were originally buried in a wooden coffin. The arrangement of the remains indicated a strong degree of decomposition of the body at the time of burial. Artifacts were also found with the remains: a buckle from the RAF pilot’s uniform, a belt buckle from the RAF pilot’s uniform, snap fasteners from the RAF pilot’s life jacket, a rubber hose and its fitting end from the RAF pilot’s life jacket, an escape compass from the RAF pilot’s equipment (Figures 3, 4). The escape compass was a very interesting find (Figure 4). Assists the pilot in escaping if captured. It was used under one of the uniform buttons. They were issued min pilots. With 10,000 RAF aid, only 30 managed to get home thanks to the compass. Due to the fact that their design was very small and primitive. Doubts were also given to their environment (Has, 2021) The compass found with the remains was a four-hole compass with a silver dial. These types of compasses were used by MI9 (British intelligence). The front glass of the compass was painted white, giving it the appearance of a standard uniform collar rivet.

Immediately after the exhumation, forensic geneticists from PBGOT collected material for genetic testing (femur fragments), described as evidence. DNA profiles obtained from the later stage of the research were deposited in the evidence base of the Polish Database of Genetic Victims Totalitarianism (PBGOT, www.pbgot.pl) and properly coded. Biological material has been secured and pre-treated under laboratory conditions following a laboratory procedure developed by our team.

The bone preparation procedure before the DNA isolation process is described in the section on genetic methods.

### 3.2 The search of possible victims

The selection of the victim’s possible identity was preceded by an analysis of historical sources and dedicated websites of the Polish Air Force (www.polishairforce.pl) and the Blue Squadron (niebieskaeskadra.pl). Historical queries conducted by MDiKN were also verified. On this basis, four pilots were selected whose bodies could be buried in the French cemetery of Le Crotoy (Table 1).

Ultimately, the list of possible victims was narrowed down to two names. F/O Tadeusz Stabrowski of No. 308 Squadron RAF, missing on 12 March 1943 in the Dieppe area, or Second Lieutenant J.S of No. 610 Squadron RAF, missing on 13 February 1943 in the Le Touquet area, or about 15 miles to the southeast, was buried there. from Boulogne by the sea.

### 3.2 Reference material

The extensive search for close relatives of all four pilots conducted by the Polish Ministry of Culture and National Heritage allowed for the relatives of all four missings. Comparative material was collected from J.G’s nephew, L.K’s niece, two nephews, and three nieces of J.S. and Tadeusz Stabrowski’s son. Finding Tadeusz Stabrowski’s son was a big problem. Tadeusz Stabrowski’s son, due to the difficult pronunciation of his surname, changed it to his stepfather’s surname. And under a changed name he emigrated to Canada. Reference material from relatives was collected in the form of swabs from the inside of the cheek on sterile swabs, sent to the laboratory, and frozen until isolation at −20°C. The DNA profiles obtained at a later stage of the research were placed in the PBGOT DNA evidence profile database and properly coded.

### 3.4 Anthropological methods

After prior reconstruction, bone material was processed according to methods commonly used in anthropology. The first stage of the work presents a detailed analysis of the examined remains. The second stage of the presented expertise is the analysis of injuries visible on the skeletons.

All anthropological research took place at the exhumation site (le Crotoy cemetery in France). For this reason, the choice of analysis methodology had to be limited to macroscopic techniques only. At the site of the works, a medical point was created where tests were carried out.

The main purpose of the research was to identify the person to whom the skeleton belonged. For this purpose, the biological profile of the remains had to be assessed. Moreover, any traumatic perimortem changes on the bones had to be analyzed to contribute to revealing the mechanism of death.

1) Biological age at death was estimated based on symphysis pubic morphology (Lovejoy, 1985), ear surface morphology (Lovejoy, 1985; Lovejoy et al., 1985), and ossification of skeletal elements (Cunningham et al., 2000). These methods were based on a comprehensive (i.e., multi-feature) analysis, taking into account changes leading to the determination of individual morphological features of the skeleton. In each of them, attention was paid to the degree of obliteration of the cranial sutures (if the preserved bone parts allow it), the state of ossification of the skeleton, any degenerative changes of the skeletal system, the degree of spongy substance compactness, the wall thickness and the height of the medullary cavities in the proximal epiphyses of the humerus (femur, humerus) changes behind the overlapping surface of the pubic symphysis and the auricular surfaces of the pelvis
2) Biological sex was estimated on the basis of pelvic morphological features (Beck et al., 1995). Particular emphasis was placed on the method (Klales and Burns, 2017), which gives results with an accuracy of over 90%. When determining the sex, attention should be paid to the metric and descriptive differentiating features in which dimorphism is visible in each of the elements (primarily, this applies to the load-bearing features of the skull bones about the so-called general morphological impression). How metric measurements of the thickness and/or a single bone of the skeleton were used, as well as the analysis of their mass.
3) Height was estimated from left femur metric scores using a regression formula adjusted for the subject’s sex and ethnicity (Trotter and Stewart, 1970)
4) Since the skeleton was preserved without the skull, the biogeographical origin could not be estimated. For the remaining components of the assessment of the biological profile, it was assumed that the person is European.
5) The state of preservation was assessed based on the quantity and quality of the preserved bones.
6) Injuries were determined based on the analysis of observed and diagnosed bone damage.

All methods used in the assessment of the biological profile are widely used in the scientific community of physical and forensic anthropologists.

### 3.5 Genetic methods

The material for genetic research was collected immediately after exhumation and properly protected against further DNA degradation.

A consensus method was used for genotyping the skeleton. It consists in obtaining three independent amplifications from one biological material taken from NN remains. In this case, there were three femur samples. To proceed to the next steps of identification, these three profiles must be identical in all tested STR systems. Bone preparation procedure before DNA isolation process.

The obtained fragment of the material is mechanically cleaned on a milling machine (Proxxon) using vanadium-tungsten cutters, then for pre-treatment of tissues until the fragment of material that is particularly exposed to putrefactive, bacterial and fungal metabolites, and DNase is removed. The material was then chemically cleaned by rinsing in Tween 20 solution for 5 min on a shaker. The next step is rinsing in distilled water. The last stage was drying the material in a laminar chamber with idle flow and exposure to UV lamp rays for 30 min on each side to remove impurities. The material prepared in this way was ground in a cryogenic mill in a liquid nitrogen environment to obtain bone powder. Bone powder was placed in sterile falcons until isolation, conserving at −80 St. C. 50 mg of bone powder was taken for DNA isolation.

#### 3.5.1 DNA isolation

Biological material taken from NN remains was isolated using the PrepFiler^®^ BTA Forensic DNA Extraction Kit (Reference, 2012). 50 mg of bone powder was used for the extraction in each case. The set is based on magnetic insulation. Lysis is performed with BTA buffer, which more easily releases DNA from calcified material. DNA extraction of comparative biological material was performed using the PrepFiler^®^ Forensic DNA extraction kit in the AutoMate Express™ DNA extraction system.

Negative and positive controls (known blood concentration on the FTA card) were used for each DNA extraction process.

#### 3.5.2 DNA concentration determination

To assess the DNA concentration and possible presence of inhibition in each extracted sample, the Quantifiler^®^ TRIO DNA Quantification Kit (Thermo Fisher Scientific, Waltham, MA, United States) was used according to the manufacturer’s instructions (Gouveia et al., 2015). DNA quantification was performed using an Applied Biosystems 7500 Real-Time PCR System (Thermo Fisher Scientific, Waltham, MA, United States).

#### 3.5.3 Amplification

DNA extracts from both evidence and control sample were amplified by Veriti^®^ 96-Well Thermal Cycler using the GlobalFiler™ PCR Amplification Kit (Thermo Fisher Scientific) which includes 24 Short Tandem Repeats (STRs) markers according to the manufacturer’s protocols (Thermo Fisher Scientific, 2019). The results are presented in Tabele 2. For the analysis of Y-STR markers, the Yfiler™ Plus PCR Amplification Kit (Applied Biosystems) was used, and the kit amplifies 25 Y-STR loci. The results are presented in Table 3.

Negative and positive PCR controls included in the kit were used.

#### 3.5.4 Detection

Detection of PCR products was performed on a 3500 Genetic Analyzer sequencer. The results were analyzed using GeneMapper^®^ ID-X Software v1.4. During data analysis, the analytical threshold, cut-off point (AT) was set at 100 RFU, as internally validated by the laboratory. Alleles below this value were not considered.

#### 3.5.5 Statistical analysis

Statistical calculations were performed using GenoProof 3.0 software (Qualitype AG, Drezno, Germany), which is software designed to perform biostatic calculations for kinship analyses, population studies, chimerism analyses, forensics, and batch studies. All types of autosomal STR scores were combined to calculate likelihood ratios for each of the two hypotheses. Mutation rates, silent alleles, and subpopulations can be used in the calculations as needed. The software used allows for more than two hypotheses in one estimate. Two mutually exclusive hypotheses were used in our study: the NN man is the son of his father, and the NN man is not the son of the putative father. The calculation of the probability of pedigree origin is based on the frequency of genotypes of persons included in the pedigree. The software used for the statistical analysis assumes an *a priori* probability of 0.5, which means that both hypotheses are equally likely. This assumption is not always true when considering near events. The frequency of rare alleles was set at 0.001. In cases where the Polish population database did not contain all GlobalFiler markers, the nearest population data - German (provided by GenoProof 3.0 software) was used.

## 4 Results

### 4.1 Anthropological results

The skeleton is preserved in an incomplete state (70%) without the skull and mandible. The condition of the bones is moderately good, with numerous traces of post-mortem erosion on the shafts of the rib bones, the shafts of the thoracic vertebrae, the epiphysis of the right distal femur, and the pubic bones. The epiphyses of the long bones are completely fused with the diaphysis. The probable estimated age by anthropological methods at the time of death was 25–35 years. The biological sex of the skeleton was determined to be male. The approximate height of the exhumed remains was about 166–174 cm.

State of preservation of the skeleton:

From the axial skeleton: all regions of vertebral column incomplete. From the cervical region no C3 and C4 vertebrae present, all remaining without significant damage and erosion. The most poorly preserved is the thoracic region, from which Th4, Th5 and Th6 are not present, from lumbar region only L4 and L5 vertebrae present. Right ribs I-X present, left ribs I-VII present, all with incomplete and damaged shafts. On a few of primal right and left ribs’ shafts perimortem injuries visible, sternum not present.

Upper extremity: bones of shoulder girdle incomplete, both scapulae and right clavicle missing. No left humerus, right humerus with damage to the proximal epiphysis. Bones of forearm from both extremities present, both radial bones complete, left ulna with damage to the proximal part of shaft—broken post-mortem, right ulna complete. Bones of the right hand complete, from the bones of the left hand only one carpal bone—capitate, three metacarpals—I-III, and one distal phalanx - from the V finger—present.

Lower extremity: pelvic bones incomplete, with erosion to the left pubis, parts of superior and inferior pubic rami missing. Right femur with damaged, preserved separately distal epiphysis, left femur with damage to the proximal epiphysis, head of the left femur preserved separately. Signs of post-mortem erosion visible on the distal epiphysis of the right femur. Bones of crus present, shaft of left fibula broken post-mortem. Tarsal bones incomplete, both calcanei, left talus and left metatarsal bones present, no phalanges. Bones on averagely massive, with averagely marked MSM (Eng. Musculoskeletal Stress Markers).

State of preservation of teeth: No teeth preserved.

Pathologies, malformations: None.

**SCHEME 1:**
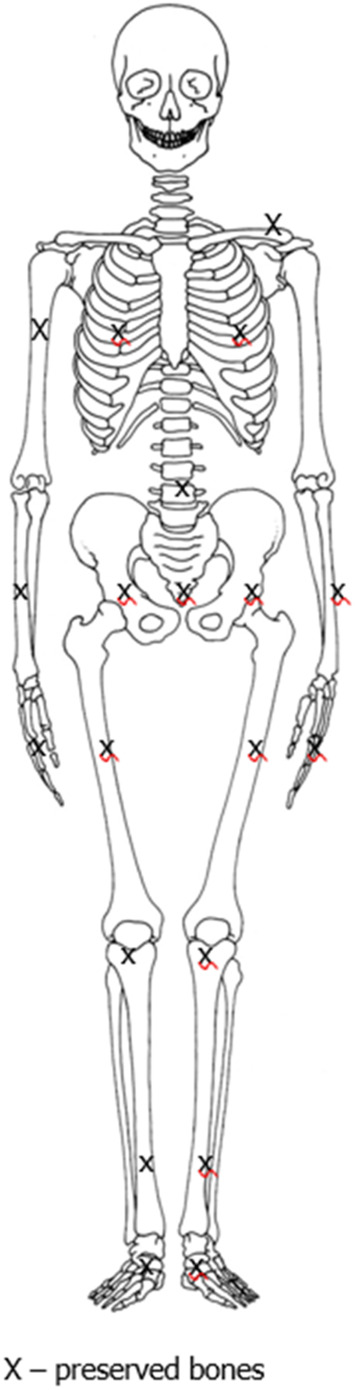
Schematic drawing of state of preservation and injuries of the skeleton.

Injuries: Marks of perimortem injuries to the shafts of ribs. Injuries to shafts of ribs, a result of perimortem fractures. Severe erosion of the distal epiphysis of the left femur (*post mortem*). The state of preservation of the skeleton and its damage are shown in Scheme 1.

When analyzing the biomechanics of the above-mentioned injuries, it should be mentioned that: so many rib fractures, especially around the vertebrae, usually occur during serious multi-organ injuries, such as traffic accidents, falls from a height, where the chest is deformed. The above-described wedge fracture of the shaft of the ulna most often occurs as a result of a direct injury acting transversely to the long axis of the forearm. Fractures of the proximal femoral epiphysis, fibula shaft, and metatarsal bones are the result of a large spiral force of the trunk on the lower limb, most often found in victims of traffic accidents or victims who fall from a height, landing on straight legs. The mechanism of the fractures found in the chest suggests that considerable pressure was applied at the moment of impact. Injury to the femur, fibula, and metatarsal means that the lower limb muscles were strained during the impact. Clearly, stronger discolorations visible on the bones are probably places where there was a massive hemorrhage and prolonged hemolysis with parts of clothing. Due to the incomplete preservation of the skeleton, a skull injury (lack of head) could not be detected. Summing up, the presence of multiple bone fractures in the described location and nature suggests that the young man’s death was caused by a high-energy multiorgan trauma, such as an airplane accident. There were no signs of a gunshot or sharp-edged injury.

### 4.2 Genetic results

A small fragment of the femoral shaft was collected for DNA isolation (approximately 5 cm). The analysis was performed in triplicate, from three independent biological samples. Identical DNA profiles were obtained from the evidence. The following human DNA concentrations were obtained from the three femur isolates: 0.01765 ng/μL; 0.016.28 ng/μL; 0.01647 ng/μL. The mean concentration of human DNA obtained was 0.016 ng/μL. This concentration of DNA was used for PCR amplification. The PCR reaction was performed in triplicate. The same results were obtained. When analyzing the obtained results, the degradation index was taken into account, which was the following: 2.300; 1.1675; 3.2195.

As a result of genetic analysis in the GlobalFiler individual identification system (Applied Biosystems), a male DNA profile was obtained from the reference material and a male DNA profile from evidence (Table 2).

**TABLE 2:** Results of allele match tests on autosomal STR markers between the consensus sample and the reference sample.

Marker	Consensus profile	Reference sample
D3S1358	16,18	C
vWA	17,19	C
D16S539	9,10	C
CSF1PO	11	C
TPOX	8,9	C
INS/DEL	2	C
AMELO	XY	C
D8S1179	10,15	C
D21S11	29	C
D18S51	14,16	C
DYS391	10	C
D2S441	10,11	C
D19S433	14,15	C
TH01	9	C
FGA	22,23	C
D22S1045	16	C
D5S818	13	C
D13S317	8,12	C
D7S820	10	C
SE33	15,19	C
D10S1248	14	C
D1S1656	15,17.3	C
D12S391	—	D
D2S1338	17,23	C

C, concordant; D, dropout of evidence material.

The results of genetic analyzes were used to verify the accepted hypothesis by determining the likelihood ratio (LR), which determines the evidentiary (statistical) power of the analyzed genetic results, analyzed in the GenoProof 3 system (quality GmbH). Analysis of the electropherograms obtained from the STR and Y-STR markers in the evidentiary samples indicates degradation of the genetic material contained in the sample, as evidenced by the decrease in peak heights with increasing length of the examined fragment (thanks to the protection of personal data of the victim’s relatives (data not shown). The obtained DNA profile for both the autosomal markers STR and Y-STR, were considered suitable for comparative testing, a result demonstrating the effectiveness of genetic methods for obtaining data that allows for comparative testing even using degraded bone material. The STR profile obtained from the DNA sample of the unidentified remains was compared with the STR profiles of all relatives who donated DNA for testing. A comparison of the STR profiles between the evidence and one of the alleged relatives showed that the results were 447,280 times more likely to be assumed if the man whose remains were to be identified as the father of his son than if they were assumed to be unrelated.

The above test results confirm with a very high probability (99.9997764%) that the NN-man is F/O Tadeusz Stabrowski, a pilot of the 308th Fighter Squadron, the so-called “Krakowiaks.”

In order to obtain the LR value for the assumed hypothesis based on the tested Y-STR markers, a search was made in the YHRD database (yhrd.org, R56, accessed on: 30.10.2017; search settings: Dataset: Y27, Kit: Y Filer Plus, Population: Eura-sian-European-Eastern European; release 56). The identified identical DNA haplotype at the Y-STR loci for the evidence sample and the reference material did not appear in the YHRD database of 8,528 samples collected from individuals representing the Eastern European population.

Based on the detailed calculations of the database search tool, it is 1,504 times more likely that an NN male is related in the male line to Andre Melaney than that there is no such relationship between them.

**FIGURE 5:**
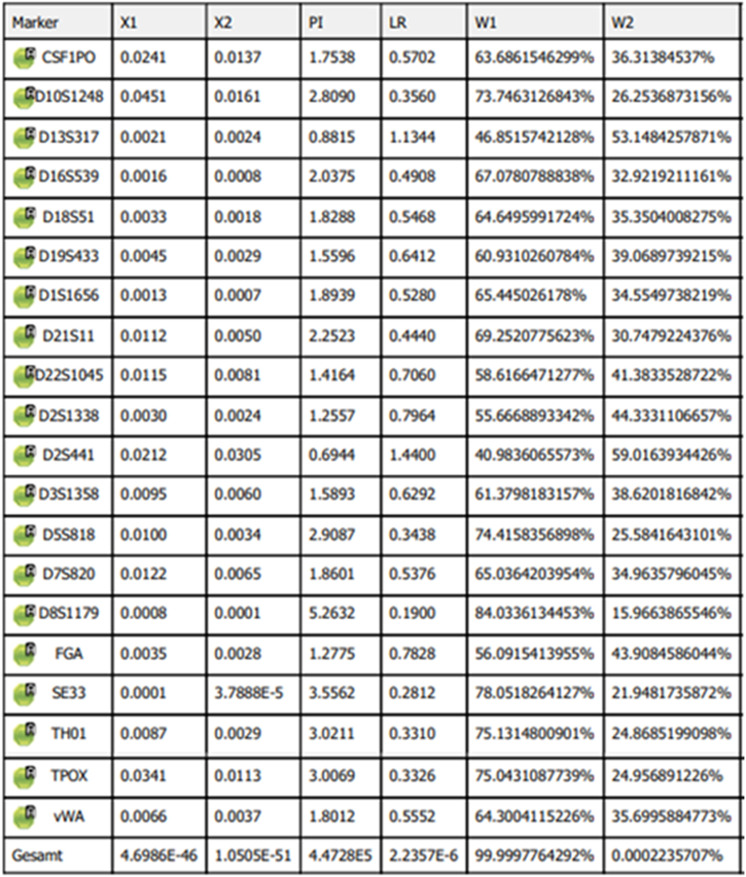
Data generated from the results of statistical calculations for Hypotheses 1 and 2 performed in GenoProof 3—source software GenoProof 3 (Qualitype GmbH).

The statistical analysis in the GenoProof 3.0 program is shown in Figure 5. Two hypotheses were taken into account in the statistical calculations: Hypothesis 1, which assumed that the NN-man is the father of his son, and Hypothesis 2, that the NN-man is not the father of his son. The figure shows the probability calculations for individual markers analyzed during the study, the total probability values for each of the adopted hypotheses (W1—hypothesis 1, W2—hypothesis 2), and the total likelihood ratio (in the program described as PI—paternity index).

Subsequent analyzes of the Y-chromosome STRs confirmed a nearly identical Y-STR profile between the evidence and the DNA from the putative son, confirming the father-son relationship (Table 3). Four Y-STR loci: DYS627, DYS391, DYS518, and DYS533 were not found in the evidence. These markers were not amplified due to the degradation of the biological material.

**TABLE 3:** The results of allele compatibility testing in Y- STR markers the consensus sample and the reference sample.

Marker	Consensus profile	Reference sample
DYS576	18	C
DYS389I	12	C
DYS635	21	C
DYS389II	28	C
DYS627	-	D
DYS460	10	C
DYS458	17	C
DYS19	15	C
YGATAH4	11	C
DYS448	19	C
DYS391	-	D
DYS456	12	C
DYS390	23	C
DYS438	9	C
DYS392	11	C
DYS518	-	D
DYS570	19	C
DYS437	15	C
DYS385	13,17	C
DYS449	28	C
DYS393	12	C
DYS439	11	C
DYS481	22	C
DYF387S1	39	C
DYS533	-	D

C, concordant; D, dropout of evidence material.

Analysis of the polymorphic DNA fragments of the control and evidence samples allowed the determination of an identical Y-STR haplotype for both samples.

Taking into account both analyzed types of markers (autosomal DNA STR mark-ers, Y-STR), it should be stated that obtaining such test results is more than 672,709,120 times more probable assuming that the identified remains belong to the father of the man from whom the reference material came than with assuming there is no relationship between them.

## 5 Discussion

Identification of human remains from several decades ago is always a challenge for the entire research team. It results mainly from the poor condition of the exhumed remains and the environmental conditions in which they decomposed, but also from the lack of reliable historical sources, access to which is often limited. Our research concerned the identification of a Polish pilot of the 308th Fighter Squadron, Lieutenant Tadeusz Stabrowski.

Markers linked to the Y chromosome significantly increase the identification possibilities of NN remains of male origin, in the case where only a relative is a person related in the male line. Our experience shows that each case should be treated individually (Ossowski et al., 2009; Kuś et al., 2015; Ossowski et al., 2016a; Ossowski et al., 2016b; Diepenbroek et al., 2019). Studies conducted in various regions of Europe (Definis Gojanović and Sutlović, 2007; Marjanović et al., 2007; PaloHedman et al., 2007; Kuś et al., 2015; Zielińska et al., 2015) show that DNA analyses appear to be the only viable approach to identifying World War II remains. For identification purposes, various DNA markers are used, which are dictated by the line of kinship. They concern autosomal markers (Marjanović et al., 2007), mitochondrial DNA (PaloHedman et al., 2007), and Y-STR (Diepenbroek et al., 2019; Grochowalski et al., 2020) Differences in the approach to identification tests may also result from the quantity and quality of the DNA obtained. It is well known that there are differences in the number of DNA templates depending on environmental conditions (PaloHedman et al., 2007) In our case, the remains were well preserved, which allowed us to obtain a good DNA template. We also had comparative material taken from the victim’s closest relative, the alleged son.

In the case of exhumation of remains from World War II and older, the amount of DNA contained in them depends on the environmental conditions in which the corpse was decomposing. Ancient and forensic DNA analysis are intertwined. Their success depends on the selection of appropriate laboratory procedures (preparation for tests, DNA isolation). The very process of securing the biological sample before it is sent to the laboratory (collection after exhumation, freezing for analysis at −20°C) is also important. (Hofreiter et al., 2021). An additional problem faced by research teams are current PCR inhibitors that affect the success of DNA typing (Alaeddini et al., 2010; Alaeddini, 2012). Such procedures are of great importance when analyzing skeletal remains in poor condition (Irwin et al., 2012). In the analyzed case, our team supervised all the work from the moment of collecting the remains, through the collection of biological material, to the laboratory work itself. The entire process was carried out in accordance with all procedures developed by our team, which allowed to avoid contamination with exogenous DNA and degradation of the material. Nowadays, research teams must bring together specialists from various fields. The case we analyzed required the cooperation of min With historians, archaeologists, anthropologists, and forensic geneticists. Without extensive analysis of historical sources, identification would be impossible. Such extremely different fields of science complement each other and provide great opportunities for future identification.

One of the most interesting elements of the above process of identification research was the use of internet tools to search for relatives in the form of portals devoted to the history of aviation from the Second World War and discussion forums for history enthusiasts and popular social networking sites. Despite the decades that have passed since the death of soldiers from the Second World War, it might seem that with the passage of time, the possibilities of identification are getting smaller due to the death of close relatives. However, the development of modern techniques gives us new opportunities for genetic analysis, which in turn gives the possibility of identification based on material from distant relatives. There are also new opportunities through the increasingly frequent sharing of archive data on the web and the use of social media that allow for quick and easy contact. Already in 2013, our Team in the work on the identification of Soviet prisoners of war showed how valuable and effective it is to obtain archival documents from online databases (Ossowski et al., 2013).

The process of identifying the victims of World War II remains very important due to their huge number and the need to honor them, for example, by restoring their identity. This is significantly hindered by the lack of archival documentation or the lack of direct access to it. Other researchers also encounter these problems (Calacal et al., 2005; Definis Gojanović and Sutlović, 2007; Marjanović et al., 2007; Ossowski et al., 2016b; Zupanič Pajnič et al., 2020). In our case, we took into account the circumstances of the victim’s death, artifacts from the RAF pilot’s vest, and documents provided by MDiKN, which allowed us to link all these elements to the victim. How important it is can be seen in the work carried out by other researchers (PaloHedman et al., 2007). Other authors around the world take a similar approach to the matter (Ríos et al., 2010; Núńez et al., 2011).

At the beginning of 1940, the Polish Air Force was operationally related to the structure of the RAF, although in various respects it was part of the independent armed forces of the Polish state. The reason was that the British Air Force had no strengths. Initially, the British were critical of the Polish pilots, explaining this fact by the fact that the Polish fighters were obsolete and belonged to the army defeated by the Germans. The Polish pilots taking part in the Battle of Britain sometimes paid with their lives (Król, 1982; Zdzisław Sawicki, 2007). They were then buried in nameless graves in nearby cemeteries. There are many Polish graves and cemeteries all over the world. Their register is kept by the Department of Cultural Heritage and War Losses at the Ministry of Culture and National Heritage. For us as forensic geneticists, it is important that none of these graves remain nameless. It is also important for the families of these victims who have not been able to recover their loved ones for years. Many of the victims were buried in mass graves, which makes identification even more difficult. How erroneous it is to base the identification process on dog tags or artifacts revealed by the victims was found out during the initial attempts to identify the victims of Kosovo (Brier and Jayanti, 2020) and Bosnia and Herzegovina (ICMP report, 2014). Such situations show that in the case of identification, DNA analyses should be relied on whenever possible. It is also the main identification method for victims of mass disasters and armed conflicts. INTERPOL takes a similar position, dividing the identification criteria into primary and secondary (INTERPOL, 2014).

In recent years, DNA analysis has significantly changed the perception of the process of restoring identity. His study is not limited in any way to the characteristic points of the body. They also do not require specific records regarding fingerprints, or medical and odontological records. The biological material may be bone tissue, which in the case of remains is easily accessible. In addition, identifications based on direct comparisons of body landmarks do not have as much statistical power as genetic testing.

DNA analysis is indisputably the main tool for identifying people. The genetic laboratory and its requirements for sample integrity, data collection, and processing should be subject to regular and rigorous quality controls to ensure the reliability of the analyses. A separate topic is an interdisciplinarity, which is strongly emphasized by the PBGOT team. Quality control of the processes from sampling in the field to delivery to the laboratory, typing combined with historical data and witness statements to narrow the circle of kinship before proceeding with costly analyzes has brought our team many identification successes (Ossowski et al., 2016a). The presented study proves that making an identification attempt, even with limited information about the remains of NN, may lead to the restoration of the name of a person who was considered missing for several years.

### 5.1 Biographical note of Tadeusz Stabrowski

A grave of an unknown Polish pilot, located in a British war section of a municipal cemetery in Le Crotoy in France, harbors remains of second lieutenant Tadeusz Stabrowski from 308 Polish fighter squadron of the Polish Air Forces in Great Britain. This is a result of genetic comparative analysis, conducted by specialists from Pomeranian Medical University in Szczecin, carried out on the grounds of a resolution signed by the Ministry of Culture and National Heritage.

On the 28th of September 2017, specialists from Szczecin University exhumed the remains of the unknown Polish pilot situated in the graveyard in Le Crotoy to examine the skeleton and the artifacts and collect samples for genetic comparative analysis. Examinations were conducted under the supervision of local French authorities, a representative of the Commonwealth War Graves Commission, in the presence of the Ministry of Culture and National Heritage worker, and a consul from the Embassy of the Republic of Poland in Paris.

Comparative genetic studies were carried out in the Department of Forensic Medicine of Pomeranian Medical University in Szczecin, where DNA from members of families of four pilots that lost their lives around Le Crotoy before the 12th of April 1943 was secured earlier.

Second lieutenant Tadeusz Stabrowski was seen for the last time by a friend from the squadron on the 11th of March 1943, when he was forced to make a water landing on the English Channel in a shot aircraft. He managed to get out from the Spitfire. Unfortunately, the seaplane of the maritime emergency services sent for him lost sight of him during the water landing approach and did not rescue it. The body of second lieutenant Tadeusz Stabrowski washed up on the French coast on the 12th of April 1943 around Le Crotoy, where it was buried as the remains of an unknown Polish pilot (Śliżewski, 2018).

The identification of the Polish Armed Forces in the West soldier, thanks to comparative DNA analysis, is an effect of the cooperation between the Ministry of Culture and National Heritage, Pomeranian Medical University in Szczecin, Embassy of the Republic of Poland in Paris, and aviation historians.

**FIGURE 6:**
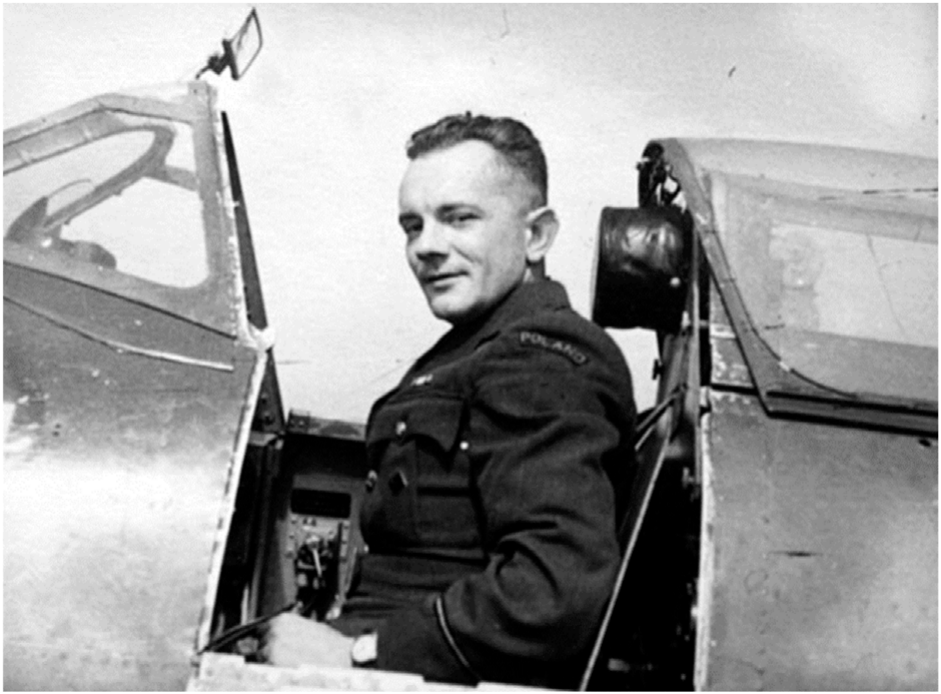
F/O Tadeusz Stabrowski in the cockpit of the Spitfire. Reproduced with permission from Wojciech Zmyślony, Second lieutenant Tadeusz Stabrowski in the cockpit of the Spitfire, http://www.polishairforce.pl/dyw308zdj.html.

Military ceremony on the graveyard in Le Crotoy, associated with restoring the full name on the pilot’s gravestone, is initially planned on the 11th of March 2018, precisely 75 years after the death of second lieutenant Tomasz Stabrowski (Figure 6).

## Data Availability

The original contributions presented in the study are included in the article/supplementary material, further inquiries can be directed to the corresponding author.
